# Asparagine Synthetase and Filamin A Have Different Roles in Ovarian Cancer

**DOI:** 10.3389/fonc.2019.01072

**Published:** 2019-10-18

**Authors:** Liang Zeng, Qiong Wang, Congmin Gu, Li Yuan, Xiaohui Xie, Lijuan He, Kai Chen, Pingping Tan, Lei Xue, Sanqian Huang, Kun Shi

**Affiliations:** ^1^Department of Pathology, Guangzhou Women and Children's Medical Center, Guangzhou Medical University, Guangzhou, China; ^2^Department of Gynecology and Obstetrics, Guangzhou Women and Children's Medical Center, Guangzhou Medical University, Guangzhou, China; ^3^Division of Uterine Vascular Biology, Guangzhou Women and Children's Medical Center, Guangzhou Institute of Pediatrics, Guangzhou Medical University, Guangzhou, China; ^4^Department of Pathology, Hunan Cancer Hospital & the Affiliated Cancer Hospital of Xiangya School of Medicine, Central South University, Changsha, China

**Keywords:** ASNS, FLNA, high-grade ovarian serous carcinoma, low-grade ovarian serous carcinoma, iTRAQ, tumor growth, cisplatin-resistance

## Abstract

Early-stage ovarian serous carcinoma is usually difficult to detect in clinical practice. The profiling of protein expression in high-grade serous carcinoma (HGSC) and low-grade serous carcinoma (LGSC) would provide important information for diagnoses and chemotherapy. Here, we performed proteomic profiling of specimens from 13 HGSC and 7 LGSC patients by iTRAQ. A total of 323 proteins that were differentially expressed were identified. After immunohistochemical confirmation of expressed proteins in 166 clinical tissues, asparagine synthetase (ASNS) and filamin A (FLNA) were selected for further functional study. Cisplatin-sensitive (CS; ASNS^high^ and FLNA^low^) and cisplatin-resistant (CR; ASNS^low^ and FLNA^high^) SKOV3 and OVCAR3 ovarian cancer cell lines were used for subsequent *in vitro* and *in vivo* experiments. Notably, ASNS overexpression (ASNS^+^) or FLNA knockdown (shFLNA) enabled cisplatin-induced apoptosis and autophagy in CR cells. However, ASNS^+^ and shFLNA promoted and attenuated tumor growth, respectively. In CS cells, ASNS knockdown (shASNS) attenuated clonogenicity, cell proliferation, and the epithelial–mesenchymal transition, whereas FLNA overexpression (FLNA^+^) protected cells from cisplatin. *In vivo*, cisplatin resistance was attenuated in mice xenografted with ASNS^+^, shFLNA, or ASNS^+^-shFLNA CR cells, whereas xenografts of shASNS or FLNA^+^ CS cells exhibited resistance to cisplatin. Clinically, all HGSC patients (83/83) responded to cisplatin, while 6 in 41 LGSC patients exhibited cisplatin resistance. These findings identify ASNS and FLNA as distinct biomarkers for HGSC and LGSC, which may have potential value in the prognosis and clinical treatment of serous carcinoma.

## Introduction

Ovarian cancer has the highest mortality rate of all female genital tract cancers (1) and poses a serious threat to women's health. Its early symptoms are not typical because of the complex endocrine function of the ovary. It is also difficult to distinguish between benign and malignant ovarian tumors prior to histological analysis. Therefore, distinction between different grades of ovarian cancer would be indispensable.

Recently, a 2-tier system to grade ovarian cancers has been validated and guidelines have been proposed for diagnosis (2). In this system, ovarian serous carcinoma is subdivided into low-grade and high-grade serous carcinoma (LGSC and HGSC) based on their genetic differences (3–5). LGSCs originate from adenofibromas or borderline tumors and are charaterized by KRAS or BRAF mutations but lack TP53 mutations (3, 6). They present low-grade nuclei with few mitotic figures and usually develop slowly with slow stepwise invasive transformation (3). LGSCs appear to be indolent, with a low probability to progress into HGSCs, and they are associated with a better prognosis than HGSCs (3). HGSCs often exhibit high levels of chromosomal instability and high-grade nuclei with frequent mitotic figures, which contribute to its more rapid growth, stronger invasiveness without prodromal lesions, but lower drug resistance to paclitaxel and carboplatin compared with LGSCs (3). HGSCs appear to originate from intraepithelial carcinoma in the fallopian tube and are characterized by TP53 mutations, BRCA germline mutations in hereditary tumors, but also the absence of KRAS or BRAF mutations (3, 4, 6). However, their differential protein profile and function, which is responsible for their phenotypic difference, are rarely reported.

In chemotherapeutics, asparagine synthetase (ASNS) can catalyze the glutamine- and ATP-dependent conversion of aspartic acid to asparagine and is involved in chemoresistance in cancer. ASNS has received considerable attention in childhood acute lymphoblastic leukemia (ALL), since increased ASNS activity in human leukemic cells is one of the causes of their resistance to L-asparaginase (7, 8). ASNS expression may regulate asparaginase resistance in extranodal natural killer (NK)/T-cell lymphoma (ENKTL) (9). In solid tumors, however, its impact on sensitivity to asparaginase has not been widely reported. In ovarian cancer cells, ASNS silencing increased asparaginase sensitivity (10, 11), which invokes the possibility in exploring ASNS as a biomarker for ovarian cancer treatment (12). In addition, ASNS contributed to doxorubicin resistance (13) and cisplatin resistance (14, 15), which presented tissue specificity of ASNS function in chemotherapeutics. Filamin A (FLNA) is a cytoskeletal protein and is possibly involved in the secretion of tissue factor-rich extracellular vesicles and DNA repair in tumors, including ovarian cancer with poor prognosis (16, 17). FLNA conferred resistance to bleomycin and cisplatin in melanoma (18) and predicted chemoresistance and poor survival in cervical cancer patients (19). Moreover, filamin A-interacting protein 1-like (FILIP1L), a key mediator of doxorubicin-induced apoptosis (20), was downregulated in ovarian cancer cell lines and clinical specimens, and negatively correlated with their invasive potential (21).

Considering distinct protein profiles is critical to diagnosis and chemotherapy. A comprehensive mass-spectrometry-based proteomic characterization of 13 ovarian HGSC and 7 LGSC specimens was performed by isobaric tags for relative and absolute quantitation (iTRAQ) technology, and two special proteins, ASNS and FLNA, were screened out for *in vitro* investigation of their function through the examination of their role in the cellular behavior of ovarian cancer cell line models.

## Materials and Methods

### Patient Population and Tissue Samples

A total of 124 ovarian cancer patients without other chronic diseases and 42 female volunteers acting as negative controls (NC) diagnosed with uterine fibroids or benign polyps at the Hunan Cancer Hospital, but who were without diabetes, hypertension, or other medication history in the last 6 months, were recruited in 2016 at the Hunan Cancer Hospital (Changsha, China). According to FIGO guidelines for ovarian carcinoma grading, 41 patients were diagnosed as LGSC while 83 patients were diagnosed as HGSC (Table 1). Written informed consent was obtained from all patients involved in this study in accordance with the Declaration of Helsinki and Good Clinical Practice guidelines. Ethical approval was obtained from the Ethics Committee of Hunan Cancer Hospital and the Ethics Committee of Guangzhou Women and Children's Medical Center. Fresh specimens of ovarian tumors were collected intraoperatively. Each specimen was divided into 3 parts: one part was for rapid diagnosis by frozen section during the operation, one part was stored in liquid nitrogen for iTRAQ proteomic examination, and one part was formaldehyde-fixed and embedded in paraffin for HE staining to identify pathological type and for immunohistochemistry (IHC) staining to confirm *in situ* expression of the differentially expressed proteins.

**Table 1:** Clinical information of LGSC and HGSC patients.

**Characteristics**	**Histology type**		***P*-value**
	**LGSC**	**HGSC**	
All cases	41	83	
Age (years)	43 ± 12	53 ± 9	0.0000
**FIGO stage** ***n*** **(%)**			
I	21 (51)	0 (0)	0.0000
II	14 (34)	8 (9)	0.0008
III	6 (15)	66 (80)	0.0000
IV	0 (0)	9 (11)	0.0286
**CA125 level in primary tumor (U/mL)** ***n*** **(%)**			
<200	2 (5)	0 (0)	0.04250
200–10,000	29 (71)	5 (6)	0.00000
>10,000	10 (24)	78 (94)	0.00000
**Ascites** ***n*** **(%)**			
Yes	13 (32)	71 (86)	0.00000
No	28 (68)	12 (14)	
**Cisplatin resistance** ***n*** **(%)**			
Yes	6 (15)	0 (0)	0.00035
No	35 (85)	83 (100)	

### iTRAQ Proteomics

#### Protein Extraction and Concentration Determination

There were several criteria for the sample collection of LGSC and HGSC for proteomics. First, the samples had to be freshly collected, pure HGSC or LGSC based on pathological diagnosis, and their combinations were not included. Secondly, the sample should have been enough for the examination (sample mass >0.5g), and without necrosis. Thirdly, before the collection, written informed consent had to be obtained from the patients.

In this study, seven LGSC cases and 13 HGSC cases were collected and used for iTRAQ proteomics. All the samples were crushed by grinding them in liquid nitrogen and then lysed in a 1.5-mL EP tube on ice with 300 μL RIPA Lysis and Extraction Buffer (Thermo Fisher Scientific, MA, USA). Each sample was sonicated by 8 pulses of 10 s with an Ultrasonic disruptor (Ningbo Scientz Biotechnology, Ningbo, China) to disrupt DNA and RNA, and they were then centrifuged for 20 min at 16,000 × *g* and 4°C. The supernatants were collected, and the determination of protein concentration was performed in each supernatant by BCA Protein Assay Kit (Sangon Biotech, Shanghai, China).

#### iTRAQ Labeling

Hundred microgram protein per sample was used for iTRAQ labeling. The prepared lysates were treated with 4 μl reducing reagent for 1 h at 60°C and then blocked by 2 μl Cysteine blocking reagent for 10 min at room temperature according to the iTRAQ kit manufacturer's instructions (AB SCIEX, CA, USA). Then, the samples were added to triethylammonium bicarbonate (TEAB) (final concentration 0.5 M) and centrifuged for 20 min at 16,000 × *g*, and the supernatants were collected (repeated three times). The samples were then digested at 37°C for 2 h with trypsin (sequencing grade modified, Promega) at a ratio of 1:200 followed by incubation overnight with trypsin at a ratio of 1:50 and TEAB (0.5 M). The next day the samples were centrifuged for 20 min at 16,000 × *g*, collected, TEAB (0.5 M) was added, and they were centrifuged again, collected, and then labeled with 8-plex iTRAQ regents (AB SCIEX, CA, USA).

#### First-Dimension Peptide Separation—High pH Reversed-Phase LC (RPLC)

The peptides were loaded onto a Gemini® 3 μm NX-C18 110 Å, LC Column 150 × 2 mm (Phenomenex, CA, USA) and submitted to mobile phase A (20 mM HCOONH_4_, 2M NaOH, pH 10) and mobile phase B (20 mM HCOONH_4_, 2 M NaOH, 80% acetonitrile, pH 10). The peptides were eluted at a flow rate of 200 μL/min and detected by Dionex Ultimate 3000 (Thermo Fisher Scientific, MA, USA) with a measurement of ultraviolet wavelength 214/280 nm. The gradient conditions were: 5% B (10 min), 15% B (5 min), 50% B (45 min), 90% B (20 min), and 5% B (10 min).

#### Second-Dimension Peptide Separation—RPLC-MS

The peptide fractions were resuspended in 0.1% formic acid (FA) and 2% acetonitrile and centrifuged for 10 min at 19,000 × g and 4°C. The supernatants were collected, injected onto 3-μm resin C18 reversed-phase column 10 cm × 100 μm (Michrom Bioresources, Auburn, CA, USA), and submitted to mobile phase A (0.1% FA, 5% acetonitrile) and mobile phase B (0.1% FA, 95% acetonitrile). The peptides were eluted at a flow rate of 300 nL/min with a linear gradient from 5 to 40% of phase B over 70 min. The separated peptide fractions were then analyzed by mass spectra dynamically in data-dependent Mode with a TripleTOF 5600 system (AB SCIEX, CA, USA). The survey scans were acquired by a mass window of 400–1,250 m/z with 250 ms activation duration at a resolution ≥30,000. MS/MS scans (≤20) were activated by each MS scan with accumulation time of precursor Ions ≥100 ms and dynamic exclusion duration 20 s at a resolution ≥15,000.

#### Data Analysis

The raw data acquired from two-dimensional LC-MS/MS was processed with AB Sciex ProteinPilot 4.0 (AB Sciex, Concord, Ontario, Canada), and protein identification and quantification were achieved by searching the UniProt database (Released in May 2014; 88,725 protein sequences). Proteomic profiles and database searching based on TripleTOF® 5600+ System (AB Sciex) and ProteinPilot 4.0 (AB Sciex) were performed following the manufacturer's recommendations. Parameters were set as follows: Unused ≥ 1.3; Credibility ≥ 95%; C.V. ≤ 0.5; AVG. ≥ 1.5 or ≤ 0.67; T.TEST with *P* < 0.05; Peptides (95%) ≥ 4. To ensure the reliability and stability of the reported data, we performed the following steps for data quality control. First, before database searching, we selected “Run False Discovery Rate Analysis” in the software AB Sciex ProteinPilot for FDR control. Second, we removed the results identified by reverse database. Third, we removed those proteins with extremely high or low ratios. Lastly, we removed those proteins with abnormal quantification between technical repetition and biological repetition. A >1.5-fold change in expression was considered different between LGSC tissues and HGSC tissues. This process was repeated three times and the average was accepted as the final result. This proteomic analysis was assisted by the FitGene BioTechnology proteomic platform (http://www.fitgene.com).

### IHC Confirmation of Protein Expression

*In situ* expression patterns of all the interesting proteins that were selected from the differentially expressed protein profiles were examined by IHC staining and scoring; in total there were 166 clinical tissues, including 41 LGSC cases, 83 HGSC cases, and 42 NC cases. All the tissues were formaldehyde-fixed and embedded in paraffin. They were collected as pathological archives from May 2012 to December 2014 in the Pathology Department of the Hunan Cancer Hospital (Changsha, China). A negative control was included by replacing the primary antibody with PBS. The immunostaining was evaluated by two independent experienced pathologists. The results of the two reviewers were compared and any discrepant scores were re-examined by both pathologists to reach a consensus score. The complete IHC score (H-score) was calculated by summing the products of the percentage of positive-stained cells (0–100) that were stained at different intensity and then multiplying them by the intensity score (0: no or marginal staining; 1: weak; 2: moderate; 3: strong), as described by Kerfoot et al. (22).

### Reagents and Cell Lines

iTRAQ kit was bought from AB Sciex (CA, USA). Cisplatin, HCOONH_4_, NaOH, polybrene and puromycin were bought from Sigma-Aldrich (Shanghai, China). Primary antibodies, including Anti-ASNS, Anti-FLNA, and Anti-β-Actin antibodies, were purchased from AbCam (Cambridge, UK). Secondary antibodies, as well as acetonitrile and FA, were bought from Thermo Fisher Scientific (MA, USA). Ovarian cancer cell lines cisplatin-sensitive (CS) SKOV3 and OVCAR3 and cisplatin-resistant (CR) SKOV3 and OVCAR3 were given as gifts by Prof. Xiaofeng Zhu (Sun Yat-sen University Cancer Center, Guangzhou, China). Before the experiments, all the cell lines were authenticated by short tandem repeat (STR) profiling by DNA sequencing and tested for mycoplasma at the Shanghai Institute for Biological Sciences, Chinese Academy of Science (Shanghai, China); they were identified as ovarian cancer cells SKOV3 (serous carcinoma) and OVCAR3 (epithelial carcinoma), respectively. Immortal ovarian surface epithelium (IOSE) cell line IOSE-80 was purchased from Guangzhou Suyan Biotechnology Co. Ltd (Guangzhou, China) and was authenticated by STR profiling at Shanghai Biowing Applied Biotechnology Co. Ltd (Shanghai, China). SKOV3 cells were cultured in McCoy's 5A Medium, and OVCAR3 and IOSE-80 in RPMI1640 medium, supplemented with 10% fetal bovine serum (FBS) and antibiotics (penicillin 100 U/mL, streptomycin 0.1 mg/mL, amphotericin B 0.25 μg/mL), and maintained at 37°C in a humidified incubator with 5% CO_2_.

CR ovarian cancer cells were established according to the following protocol: CS cells were thawed and cultured at 37°C in a humidified incubator with 5% CO_2_ until ~90% confluence and then passaged. When the cells were in the exponential growth phase, 0.125 μg/mL of Cisplatin was administrated and the cells cultured until ~90% confluence. The cells were then passaged and grown until the exponential phase, and 0.25 μg/mL Cisplatin was then administrated. According to this protocol, the cells were passaged and cultured, and they were then treated with increasing concentrations of Cisiplatin upto 2 μg/mL. The ovarian cancer cells induced by this protocol were named CR ovarian cancer cells. The resistance index (RI) = IC_50_ (CR)/IC_50_ (CS) was then calculated, where IC_50_ is short for 50% inhibitory concentration. The RI for CR SKOV3 was 9.76 and for CR OVCAR3 was 6.18.

### Gene Edited Cell Lines

Full-length human ASNS and FLNA cDNA (accession numbers NM133436.3, NM_001456.3) were amplified in DH5α cells (Invitrogen, CA, USA), cloned into lentivirus vectors LV011-pHBLV-CMV-MCS-3FLAG-EF1-T2A-Zsgreen-Puro (Hanbio Biotechnology, Shanghai, China) to construct an overexpression vector, and noted as ASNS^+^ and FLNA^+^ vectors, respectively. Short hairpin RNAs (shRNAs) that selectively targeted ASNS sequence (shASNS1-1: 5′-GCTGTATGTTCAGAAGCTAAA-3′), (shASNS1-2: 5′-GCACGCCCTCTATGACAATGT-3′), (shASNS1-3: 5′-GCCATTACTGGATGCCCAAGT-3′) or targeted FLNA sequence (shFLNA2-1: 5′-GCCCACCCACTTCACAGTAAAT-3′), (shFLNA2-2: 5′-GCTGGCAGCTACACCATTATG-3′), (shFLNA2-3: 5′-GGACATCATCGACCACCATGA-3′) were amplified, cloned into lentivirus vector pHB-U6-MCS-CMV-ZsGreen-PGK-Puro (Hanbio Biotechnology, Shanghai, China) to construct knockdown vectors, and noted as shASNS and shFLNA plasmid, respectively. 293T cells were then transfected with pSPAX2, pMD2G, and the overexpression plasmid (1 μg/ml) or the knockdown plasmid (1 μg/ml) using Lipofiter™ reagent (Hanbio Biotechnology, Shanghai, China) to produce lentiviral particles. After 48 and 72 h, respectively, the supernatants of lentiviral particles were collected and centrifuged for 10 min at 2,000 × g and 4°C to discard cellular debris, followed by supernatant collection and then centrifuged for 2 h at 82,700 × g and 4°C, and finally the concentrated lentiviral particles were harvested. After viral titer examination (1 × 10^8^ TU/mL), the lentiviral particles of shASNS or FLNA^+^ were mixed with culture media (1:1) and polybrene (8 μg/ml) respetively to infect CS cells, including SKOV3-CS and OVCAR3-CS. The lentiviral particles of ASNS^+^, shFLNA or ASNS^+^+shFLNA were used to infect CR cells, including SKOV3-CR and OVCAR3-CR, respectively. All the infected cells were selected with puromycin treatment (1.5 μg/mL) for 5 days.

### Clonogenic Assay

Cells were plated at 500 cells/well in 6-well plates and CS-vector cells were used as a control. When the clones reached 50 cells/clone in the CS-vector well (7–10 days), the colonies were fixed and stained with 1.5 ml of 6.0% glutaraldehyde and 0.5% crystal violet (Sigma-Aldrich, Shanghai, China) for 2 h at room temperature, rinsed in water and counted by GelCountTM (Oxford Optronix, Oxford, U.K.). The surviving fraction (SF) of cells was calculated as follows: SF = $\frac{Number\;of\;colonies\;formed\;after\;treatment\;}{Number\;of\;cells\;seeded\;x\;Plating\;Efficiency\;}\;$, where Plating Efficiency = $\frac{Number\;of\;colonies\;formed\;in\;control}{Number\;of\;cells\;seeded}\;$ (23).

### Proliferation Assay

A cell proliferation assay was performed by Cell Trace carboxyfluorescein succinimidyl ester (CFSE) staining (Invitrogen, Carlsbad, CA, USA) with the protocol described previously (24).

### Invasion Assay

Cell invasion was examined by Matrigel-coated transwell assays with the protocol described previously (25).

### Migration Assay

Cell migration was examined by wound healing assays with the protocol described previously (25).

### Apoptosis Assay

Cell apoptosis assays were performed by Annexin-V/propidium iodide (PI) staining (BioLegend, San Diego, CA, USA) with the protocol described previously (24).

### Autophagy Assay

Cell autophagy assays were performed by monodansylcadaverine (MDC) staining (Sigma-Aldrich, Shanghai, China) with the protocol described previously (24).

### Cell Viability Assay

Cell viability was examined by alamarBlue staining assay (Bio-Rad, Hercules, USA) with the protocol described previously (24).

### Real-Time Quantitative PCR

Total RNA was isolated by RNAiso Plus (Takara, Dalian, China) and reverse transcribed by PrimeScript RT Master Mix (Takara). The specific primer pairs for those genes were designed below and according to the published sequence in NCBI:

ASNS: forward 5′- AGAGATTCTCTGGCGACCAAAAGA-3′, reverse 5′- CTGGGTAATGGCGTTCAAAGACTT-3′;

FLNA: forward 5′- GGGCAAATACGTCATCTGTG-3′, reverse 5′- AGGGGATGACAAGGTCAAAG-3′;

ACTB (control): forward 5′- ACAGAGCCTCGCCTTTGC-3′, reverse 5′- GCGGCGATATCATCATCC-3′.

Realtime RT-PCR was performed with the CFX96 Real-Time System (Bio-Rad, Hercules, USA). Data were analyzed and exported by Sequence Detection Software for the value of the threshold cycle (Ct), and the comparative Ct (ΔΔCt) was used to calculate the difference between samples by relative quantification (fold change).

### Western Blotting Assay

Cell lysates, after protein determination by Bio-Rad protein assay (Bio-Rad, Hercules, USA), were submitted to western blotting assay with the protocol described previously (24).

### *In vivo* Chemoresistance Examination

For *in vivo* establishment of tumor xenograft, female BALB/c nude mice (6–7 weeks old and weight 18–22 g) (Slac Laboratory Animal, Shanghai, China) were inoculated subcutaneously with ovarian cancer cells (1 × 10^7^) of the same passage number at one front flank and tumor growth was observed in the following weeks. When the tumor grew up to 100 mm^3^, the mice were randomized into different treatment groups and a control group using randomized block design based upon their tumor volumes. Tumor sizes were measured once per week in two dimensions using a caliper, and the volume (V) was expressed in mm^3^ using the formula: V = 0.5a × b^2^ where a and b were the long and the short diameters of the tumor, respectively. Seven groups were assigned (5 mice/group) according to the ovarian cancer cells used for the inoculation, which included CS-Vector cells, CS-shASNS cells, CS-FLNA^+^ cells, CR-Vector cells, CR-ASNS^+^ cells, CR-shFLNA cells, and CR-ASNS^+^-shFLNA cels. Cisplatin treatment (5 mg/kg) was performed twice in 1 week through tail vain injection administration. Six weeks later, the mice were euthanized by cervical dislocation. The animal work was approved by the Institutional Animal Care and Use Committee of Guangzhou Medical University and was conducted in accordance with the *Guide for the Care and Use of Laboratory Animals* (NIH Publication 85–23, revised 1996).

### Statistics

The difference in clinical characteristics between LGSC and HGSC patients, including the distribution of FIGO stage (I-IV) and CA125 level in the primary tumor (<200, 200–10,000, >10,000 U/mL), patients with ascites (yes/no) or with cisplatin resistance (yes/no) were analyzed with a χ2 test. In iTRAQ proteomic identification, the detection in all samples was run twice, and data analysis was performed by FDR test with the software AB Sciex ProteinPilot to confirm the reliability and stability of the reported data. Then, differences in protein expression were analyzed by Student's *t*-test. In IHC examination, complete H-scores were analyzed by Mann-Whitney *U*-test (GraphPad Prism 6, GraphPad, La Jolla, USA). Data obtained from *in vitro* examination were analyzed by one-way ANOVA followed by Turkey's *post-hoc* test (GraphPad Prism 6). Each independent experiment in IHC and *in vitro* examination was performed in triplicate and repeated three times. Data are presented as mean ± SD and *P* < 0.05 was considered statistically significant.

## Results

### Differential Protein Profiles of LGSC and HGSC

**Figure 1:**
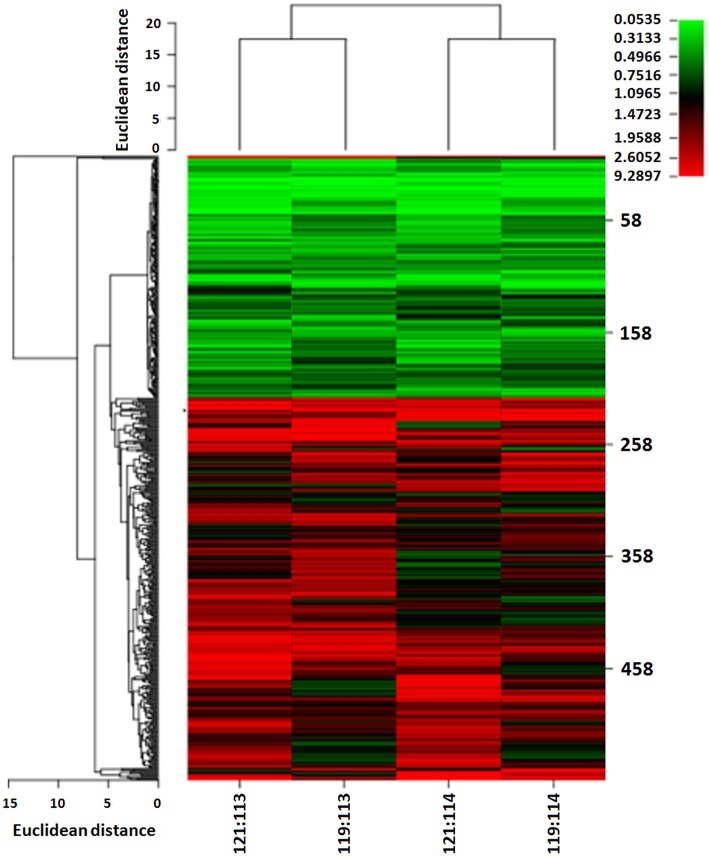
Heat map of differentially expressed proteins between LGSC and HGSC tissues examined by iTRAQ quantitative proteomics. HGSC groups (*n* = 13) were labeled as 113 and 114; LGSC groups (*n* = 7) were labeled as 119 and 121.

We used iTRAQ technology to detect differentially expressed proteins between 13 ovarian HGSC and 7 LGSC samples. The HGSC samples were formed into groups of 113 and 114, while the 7 LGSC samples were formed into groups of 119 and 121. The differentially expressed proteins are presented in Figure 1. A total of 4,964 credible proteins (95% confidence interval, unused score ≥1.3) were identified (raw data are shown in Tables S1–S4). We screened 323 proteins exhibiting evident alterations in expression, using cutoff HGSC-to-LGSC expression ratios of ≥2 and ≤0.5 to define upregulated and downregulated proteins, respectively (Table S5). The top 50 upregulated and top 50 downregulated proteins in HGSCs relative to LGSCs were selected and are shown in Tables S6, S7. Next, a gene ontology (GO) analysis was performed to classify the proteins according to their biological processes, cellular components, and molecular functions with the aim of understanding the associated molecular and functional characteristics. These proteins involved 26 cellular components, 64 biological processes, and 35 molecular functions.

Moreover, a KEGG pathway analysis revealed associations of these proteins with 197 signaling pathways, of which the following were identified as the top 10: the metabolic pathway, focal adhesion, cell-cell connections, proteoglycan in carcinoma, cell extracellular matrix receptor interaction, complement and coagulation cascades, the PI3K-Akt signal pathway, dilated cardiomyopathy, arrhythmogenic right ventricular cardiomyopathy, and amoebiasis.

### Verification of Expression of the Identified Proteins

**Figure 2:**
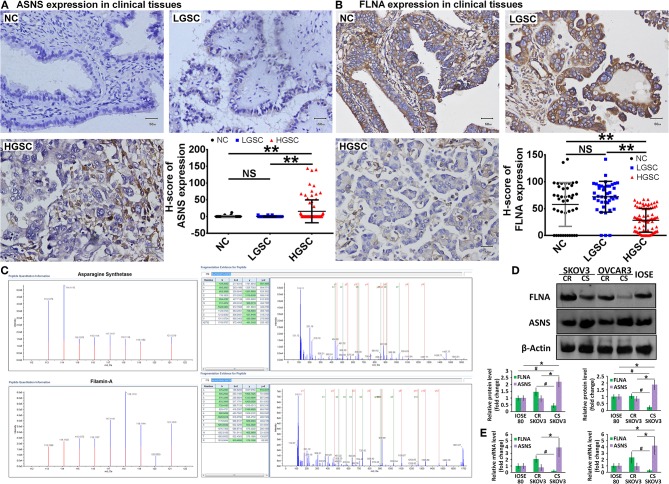
Representative images of *in situ* expression of ASNS and FLNA in NC (*n* = 42), LGSC (*n* = 41), and HGSC tissues (*n* = 83), as well as their H-score analyses **(A,B)**. Structural identification of ASNS and FLNA by mass spectrometry **(C)** (HGSC groups: *n* = 13; LGSC groups: *n* = 7). Expression of ASNS and FLNA protein **(D)** and mRNA **(E)** in CS-, CR-SKOV3 cells, CS-, CR-OVCAR3 cells, and IOSE-80 cells (negative control). Relative protein expression level or mRNA level are both shown as the level relative to the control (mean ± SD). ^#^ and * indicates the significant difference in the expression of FLNA and ASNS, respectively, in CS cells when compared with CR or IOSE-80 cells. ** indicates the significant difference in the expression of ASNS and FLNA, respectively, in HGSC group when compared with LGSC or NC group. ^#^ or *P < 0.05, **P < 0.01.

According to the GO and KEGG analyses, we selected 5 differently expressed proteins between HGSC and LGSC: guanylate-binding protein-1, stathmin-1, ASNS, retinol-binding protein-1, and FLNA. After subjecting 166 clinical samples to immunohistochemical examination, we only confirmed the differential expression of ASNS and FLNA (Figures 2A,B). Specifically, we observed higher ASNS levels and lower FLNA levels in HGSC tissues relative to LGSC or NC tissues. We did not observe differences in the expression of either protein between LGSC and NC tissues. We then used mass spectrometry to verify the structures of ASNS and FLNA (Figure 2C).

The ovarian cancer cell lines SKOV3 and OVCAR3 were used as models for the *in vitro* investigation of protein function, and the levels of ASNS and FLNA proteins and mRNAs were examined by western blotting (Figure 2D) and RT-PCR (Figure 2E), respectively. Compared with cisplatin-resistant (CR) cells, cisplatin-sensitive (CS) cells expressed higher levels of ASNS and lower levels of FLNA, which exhibited concordant differences in ASNS or FLNA expression between HGSC and LGSC, as confirmed above. The CR cells did not differ from IOSE-80 normal ovarian epithelial cells (negative control) in terms of ASNS or FLNA expression, whereas CS cells expressed relatively higher and lower levels of ASNS and FLNA, respectively.

LGSC and HGSC tissues also differed significantly in terms of cisplatin resistance (*P* = 0.00035). Six of 41 patients with LGSC in our cohort exhibited cisplatin resistance, compared to 0 of 83 patients with HGSC (Table 1). Given the concordant differences in ASNS and FLNA expression together with the differences in cisplatin resistance, we selected CS (SKOV3-CS, OVCAR3-CS) and CR cells lines (SKOV3-CR, OVCAR3-CR) as cell models for an *in vitro* examination of ASNS and FLNA function.

### Role of ASNS and FLNA in Biological Behavior of Ovarian CR and CS Cells

**Figure 3:**
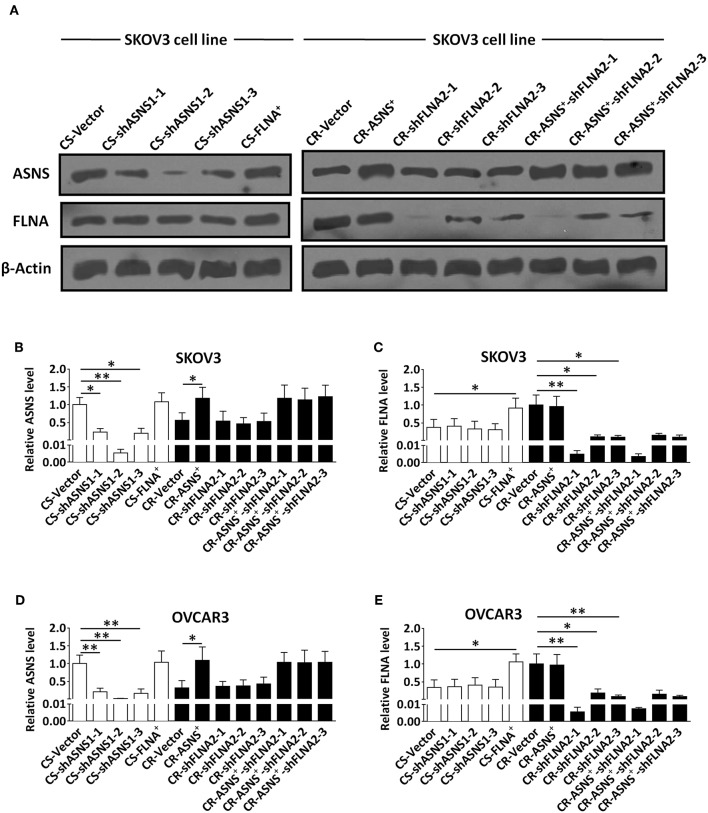
Verification of knockdown of ASNS expression and overexpression of FLNA in CS cells, as well as overexpression of ASNS and knockdown of FLNA expression in CR cells **(A–E)**. The verification was performed in CS-SKOV3, CR-SKOV3, CS-OVCAR3, and CR-OVCAR3 cells by western blotting assay. Cells with overexpression of ASNS or FLNA were constructed by ASNS^+^ or FLNA^+^ plasmid; cells with knockdown of ASNS or FLNA expression were constructed by three shRNAs for each gene in order to confirm its efficacy. * or ** indicates the significant difference in relative protein expression level (ASNS or FLNA) between the groups. *P < 0.05, **P < 0.01.

To investigate the roles of ASNS and FLNA in the behaviors of CS and CR ovarian cancer cells, we subjected CS cells to ASNS knockdown or FLNA overexpression and CR cells to ASNS overexpression or FLNA knockdown. CR cells were also subjected to simultaneous ASNS overexpression and FLNAknockdown. All manipulations of gene expression in SKOV3 and OVCAR3 cells were verified by western blotting (Figure 3).

#### ASNS Contributed to Clonogenic Growth and Cell Proliferation in CS and CR Ovarian Cancer Cells

**Figure 4:**
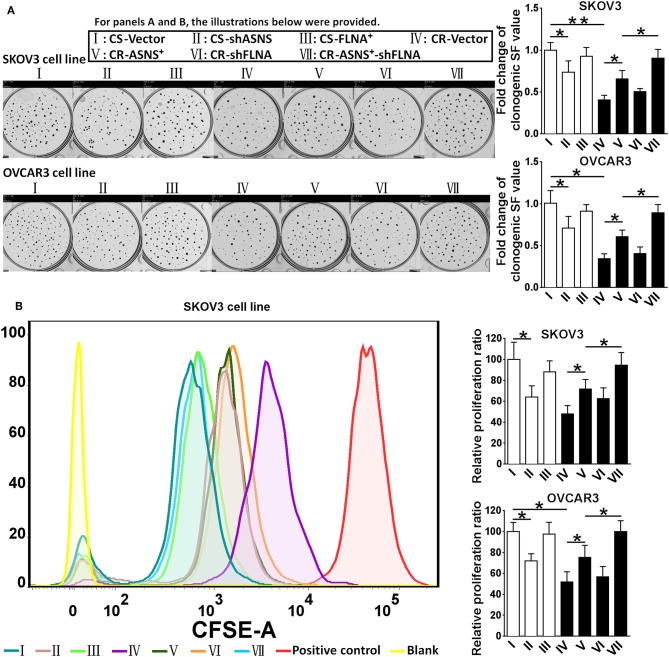
Examination of clonogenic ratio **(A)** and cell proliferation **(B)** in CS and CR cells, specifically in: CS-Vector cells, CS-shASNS1-2 cells, CS-FLNA^+^ cells, as well as in CR-Vector cells, CR-ASNS^+^ cells, CR-shFLNA2-1 cells, and CR–ASNS^+^-shFLNA2-1 cells. CS- or CR-cells include CS- or CR-SKOV3 cells and CS- or CR-OVCAR3 cells. Fold change of clonogenic SF value or relative proliferation ratio are shown as the value relative to the control (mean ± SD). * or ** indicates the significant difference in clonogenic SF value or relative proliferation ratio between the groups. *P < 0.05, **P < 0.01.

CS cells had a higher clonogenic ratio (2.5 fold in SKOV3 cells vs. 2.9 fold in OVCAR3 cells; Figure 4A) and a higher proliferation ratio (2.1 fold in SKOV3 cells vs. 1.9 fold in OVCAR3 cells; Figure 4B). ASNS knockdown significantly reduced the clonogenic and proliferation ratios in CS cells, whereas ASNS overexpression significantly increased both ratios in CR cells (clonogenic ratio: 1.5 fold in SKOV3 vs. 1.8 fold in OVCAR3 cells; proliferation ratio: 1.5 fold in SKOV3 vs. 1.3 fold in OVCAR3 cells). In contrast, FLNA overexpression or knockdown did not alter the clonogenic or proliferation ratios in CS or CR cells. The combination of FLNA knockdown and ASNS overexpression further upregulated the clonogenic and proliferation ratios of CR cells when compared to ASNS overexpression alone.

#### ASNS Promoted the Epithelial–Mesenchymal Transition (EMT) in CS and CR Ovarian Cancer Cells

**Figure 5:**
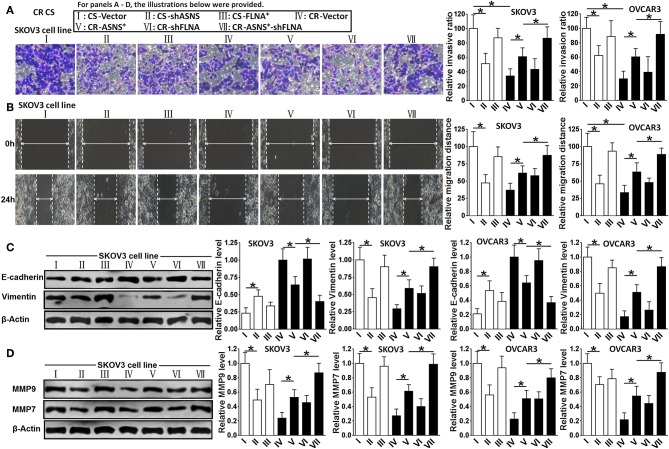
Examination of invasion **(A)**, migration **(B)**, expression of E-cadherin and Vimentin **(C)**, and expression of MMP-9 and MMP-7 **(D)** in CS and CR cells, specifically, in CS-Vector cells, CS-shASNS1-2 cells, CS-FLNA^+^ cells, as well as in CR-Vector cells, CR-ASNS^+^ cells, CR-shFLNA2-1 cells, and CR–ASNS^+^-shFLNA2-1 cells. CS- or CR-cells include CS- or CR-SKOV3 cells and CS- or CR-OVCAR3 cells. Relative invasion ratio, relative migration ratio, and relative expression level of E-cadherin, Vimentin, MMP-9, or MMP-7 are shown as the value relative to the control (mean ± SD). * indicate the significant difference between groups in relative invasion ratio, relative migration distance, relative expression level of E-cadherin, Vimentin, MMP-9 or MMP-7. *P < 0.05.

Compared with CR cells, CS cells were more invasive (2.8 fold in SKOV3 cells and 3.4 fold in OVCAR3 cells) and more migratory (2.5 fold in SKOV3 cells and 3.1 fold in OVCAR3 cells; Figures 5A,B). CS cells also expressed relatively higher levels of vimentin, MMP-9, and MMP-7 as well as a lower level of E-cadherin (Figures 5C,D). In CS cells, ASNS knockdown attenuated cell invasion and migration; enhanced E-cadherin expression; and decreased vimentin, MMP-9, and MMP-7 expression. In CR cells, ASNS overexpression promoted cell invasion (1.7 fold in SKOV3 cells and 2.2 fold in OVCAR3 cells) and migration (1.7 fold in SKOV3 cells and 1.9 fold in OVCAR3 cells) and decreased E-cadherin expression. Neither FLNA overexpression nor knockdown affected invasiveness, migratory behavior, or EMT marker expression in CS or CR cells. However, the combination of FLNA knockdown and ASNS overexpression more strongly promoted invasion, migration, and EMT marker expression in CR cells when compared to ASNS overexpression alone.

#### ASNS Inhibited Autophagy and Promoted Apoptosis, Whereas FLNA Had Suppressed Both Processes

**Figure 6:**
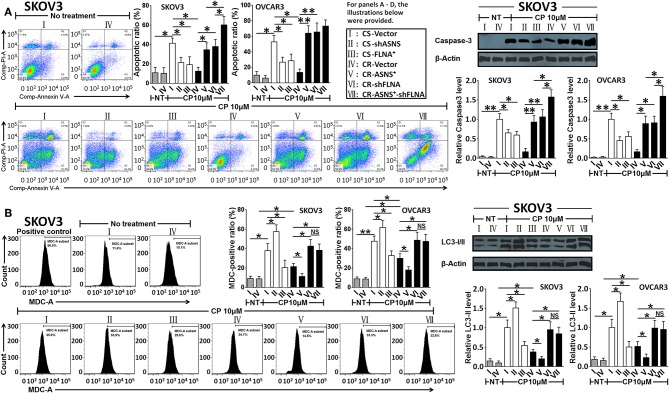
Examination of apoptosis **(A)** and autophagy **(B)** after 72 h-treatment with cisplatin in CS and CR cells, specifically, in CS-Vector cells, CS-shASNS1-2 cells, CS-FLNA^+^ cells, as well as in CR-Vector cells, CR-ASNS^+^ cells, CR-shFLNA2-1 cells and CR–ASNS^+^-shFLNA2-1 cells. CS- or CR-cells include CS- or CR-SKOV3 cells and CS- or CR-OVCAR3 cells. Apoptotic ratio and MDC-positive ratio are shown as mean ± SD. Relative expression levels of cleaved-Caspase-3 or LC3-II are shown as the level relative to the control (mean ± SD). * and ** indicate the significant difference between groups. *P < 0.05, **P < 0.01.

After a 72-h treatment with cisplatin (10 μM), CS cells exhibited a higher apoptotic ratio and caspase-3 activation level (Figure 6A) as well as an increased MDC (autophagy indicator)-positive ratio and LC3-II level (Figure 6B) when compared with CR cells (apoptotic ratio: 5.0 fold in SKOV3 and 3.9 fold in OVCAR3 cells; MDC-positive ratio: 1.8 fold in SKOV3 and 1.5 fold in OVCAR3 cells). In CS cells, ASNS knockdown significantly reduced cisplatin-induced apoptosis and increased autophagy (1.5 fold in SKOV3 vs. 1.2-fold in OVCAR3 cells). In CR cells, ASNS overexpression promoted cisplatin-induced apoptosis (2.6 fold in SKOV3 and 5.6-fold in OVCAR3 cells) and downregulated autophagy. In CS cells, FLNA overexpression protected against cisplatin-induced apoptosis and autophagy (Figures 6A,B). In CR cells, FLNA knockdown enhanced apoptosis (2.9 fold in SKOV3 and 5.7 fold in OVCAR3 cells) and autophagy in response to cisplatin treatment (2.0 fold in SKOV3 and 1.6 fold in OVCAR3 cells). In SKOV3-CR cells, the combination of ASNS overexpression and FLNA knockdown enhanced apoptosis to a greater extent than ASNS overexpression or FLNA knockdown alone (Figure 6A). Interestingly, although ASNS overexpression inhibited autophagy whereas FLNA knockdown enhanced autophagy in both SKOV3-CR or OVCAR3-CR cells, the combination of ASNS overexpression and FLNA knockdown enhanced autophagy relative to the level observed in CR-vector cells (Figure 6B).

#### ASNS Sensitized Cells to Cisplatin, Whereas FLNA Contributed to Cisplatin Resistance

**Figure 7:**
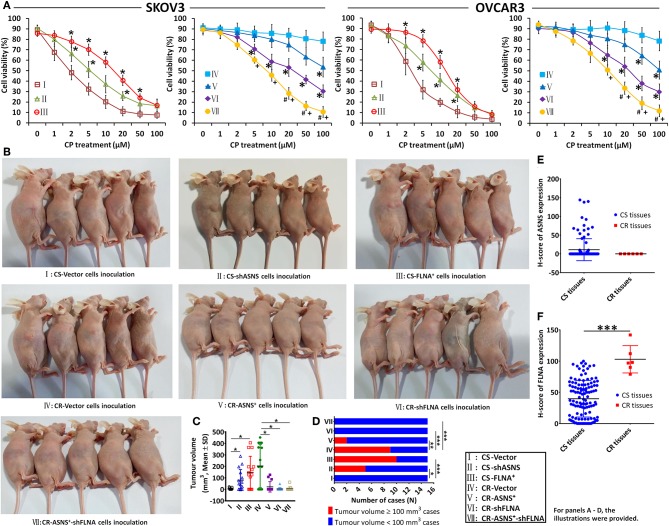
*In vitro* and *in vivo* examination of cisplatin sensitivity. Cell viability after 72 h-treatment with cisplatin was examined in CS and CR cells, specifically in CS-Vector cells, CS-shASNS1-2 cells, CS-FLNA^+^ cells, as well as in CR-Vector cells, CR-ASNS^+^ cells, CR-shFLNA2-1 cells, and CR–ASNS^+^-shFLNA2-1 cells **(A)**. CS- or CR-cells include CS- or CR-SKOV3 cells and CS- or CR-OVCAR3 cells. Cell viability is shown as mean ± SD. * indicates the significant difference in the groups compared with their Vector cell group. + indicates the significant difference in CR–ASNS^+^-shFLNA2-1 cell group compared with CR-ASNS^+^ cell group. ^#^indicates the significant difference in CR–ASNS^+^-shFLNA2-1 cell group compared with CR-shFLNA2-1 cell group. Female BALB/c nude mice were subcutaneously innoculated with CS-Vector cells, CS-shASNS cells, CS-FLNA^+^ cells, CR-Vector cells, CR-ASNS^+^ cells, CR-shFLNA cells, and CR-ASNS^+^-shFLNA cells **(B)**. After 6-week cisplatin administration, tumor volumes of all the xenografted mice were calculated **(C)**, and the tumor volume ≥ 100 mm^3^ cases as well as the tumor volume < 100 mm^3^ cases were analyzed **(D)**. *n* = 15 for each group. *, **, and *** indicate the significant difference between groups. The expression of ASNS **(E)** and FLNA **(F)** was compared with clinical CS cancer tissues (*n* = 118) and CR cancer tissues (*n* = 6). *** indicate the significant difference between groups. *, +, or ^#^*P* < 0.05, **P < 0.01, ***P < 0.001.

CS cell viability was inhibited by cisplatin (treatment with 0–100 μM for 72 h) in a dose-dependent manner, whereas the same effect was not evident in CR cells (Figure 7A). In CS cells, ASNS knockdown increased cell viability after cisplatin treatment. In CR cells, ASNS overexpression upregulated cell death in response to cisplatin treatment. In CS cells, FLNA overexpression enhanced resistance to cisplatin-induced cell death, whereas in CR cells FLNA knockdown reduced cell viability relative to the level observed in CR-vector cells. In CR cells, the combination of ASNS overexpression and FLNA knockdown enhanced sensitivity to cisplatin-induced cell death relative to ASNS overexpression or FLNA knockdown alone.

We also examined the effects of ASNS and FLNA on cisplatin sensitivity *in vivo*. Female BALB/c nude mice were xenografted with different ovarian cancer cell lines via subcutaneous inoculation (Figure 7B), and this was then followed by a 6-week course of cisplatin administration. After treatment, the average tumor volumes of CS-shASNS (79.8 mm^3^) and CS-FLNA^+^ xenografts (146.8 mm^3^) were significantly greater than the average volume of CS-vector xenografts (3.6 mm^3^; Figure 7C). Moreover, the CS-shASNS (*n* = 5) and CS-FLNA^+^ (*n* = 10) groups had significantly higher numbers of tumors with volumes ≥100 mm^3^, compared to the CS-vector group (*n* = 0; Figure 7D). Meanwhile, the average tumor volumes in the CR-ASNS^+^ (21.4 mm^3^), CR-shFLNA (3.2 mm^3^), and CR-ASNS^+^-shFLNA groups (5.1 mm^3^) were smaller than the average volume in the CR-vector group (202.7 mm^3^). The CR-ASNS^+^ (*n* = 2), CR-shFLNA (*n* = 0), and CR-ASNS^+^-shFLNA groups (*n* = 0) had significantly lower numbers of xenografts with volumes ≥100 mm^3^ when compared with the CR-vector group (*n* = 9).

Finally, we compared the expression of ASNS and FLNA in clinical tissue samples of CS (83 HGSC + 35 LGSC) and CR cancers (6 LGSC). Compared with CS tissues, CR tissues expressed high levels of FLNA and almost undetectable levels of ASNS (Figures 7E,F).

## Discussion

Ovarian LGSCs and HGSCs arise from two different types of ovarian serous carcinomas (3) via distinct and generally independent pathways. Typically, even recurrent LGSCs retain the characteristics of low-grade tumors and rarely progress to HGSCs (3). In one report, only 2% of HGSCs were associated with serous borderline tumors (26). To date, several biomarkers, such as p53, p16, and Ki-67, have been identified as useful for distinguishing between ovarian LGSC and HGSC and are useful in some settings. However, these biomarkers are of limited value (3, 4). Therefore, an understanding of the differential expression of proteins between LGSC and HGSC, and the potential functions of these proteins, would clarify the pathologic and histologic features of these tumors and may contribute to diagnostic and therapeutic approaches. In this study, we subjected LGSC and HGSC to iTRAQ proteomics and selected the differentially expressed proteins ASNS and FLNA for a functional assessment.

To date, several *in vitro* models of ovarian HGSC or LGSC have been reported. In this work, we used CS and CR ovarian cancer cell lines as *in vitro* models, but not as models of HGSC or LGSC. First, we observed a significant difference in cisplatin resistance between LGSC and HGSC patients in our cohort, like the fact that the former were more likely to exhibit increased cisplatin resistance. Clinical evidence has demonstrated the responsiveness of HGSCs to first-line (taxane- and platinum-based) chemotherapy, whereas varied responses are observed in LGSCs (3, 4, 27). In an *in vitro* investigation into drug resistance, LGSCs more frequently exhibited extreme chemoresistance to paclitaxel and carboplatin when compared with HGSCs (28). Second, we observed similar fold changes in ASNS and FLNA expression between CS and CR cells and between HGSC and LGSC samples. We note that even CR cases are limited to a subset of LGSCs. Most LGSCs and all HGSCs are CS, and therefore it is difficult to correlate cisplatin sensitivity with the tumor grade directly. Moreover, HGSC, and LGSC are distinct diseases with different origins and mutations, and these different responses to chemotherapy may therefore be unsurprising. However, these etiological differences make it difficult to consider two sublines (CS and CR) of the same parent cell line as distinct HGSC and LGSC models. To overcome this limitation, we selected cells expressing high ASNS and low FLNA levels to model CS ovarian cancers and cells expressing low ASNS and high FLNA levels to model CR ovarian cancers.

Despite this distinction, ASNS, and FLNA may attenuate and enhance cisplatin resistance in HGSC and LGSCs, respectively. This concept was inferred based on *in vitro* and *in vivo* examinations and is concordant with the characteristics of tumors observed in our patient cohort. We note that all patients with HGSC (ASNS^high^ and FLNA^low^) in our cohort (83/83) responded to cisplatin, whereas 6 of 41 patients with LGSC (ASNS^low^ and FLNA^high^) exhibited cisplatin resistance. This difference between groups was significant. A further analysis revealed strong FLNA expression in CR cancer tissues. Moreover, mice xenografted with shASNS or FLNA^+^ CS cell lines exhibited cisplatin resistance, whereas mice xenografted with ASNS^+^, shFLNA, or ASNS^+^-shFLNA CR cell lines were significantly more sensitive to cisplatin. These results confirmed our inferences from the results of CS and CR cell experiments.

The biological functions of ASNS and FLNA with respect to tumor growth and chemosensitivity have been investigated previously. Briefly, ASNS is an amidotransferase that synthesizes L-asparagine in eukaryotic cells (7). A deficiency in functional ASNS may block the synthesis of nascent peptides, inhibit the cell cycle, and trigger apoptosis (29, 30). Clinically, ASNS expression was shown to correlate with an advanced tumor grade and poor prognosis in patients with solid tumors (e.g., glioma) and blood cancers (e.g., ALL) (30, 31). In our study, ASNS overexpression enhanced clonogenicity, cell proliferation, invasion, migration, and EMT, suggesting a potential role for this protein in cancer growth and metastasis. ASNS overexpression also inhibited autophagy and promoted cisplatin-induced apoptosis, whereas ASNS knockdown stimulated autophagy and reduced apoptosis. Liu et al. reported the upregulation of ASNS and MMP-19 in CS S16 nasopharyngeal carcinoma cell cells, relative to CNE-2 cells (parental cells of S16). Cisplatin sensitivity was conferred on S16 cells by suppressing the expression of nucleotide excision repair (NER) genes (e.g., Rad23B, RPA32, XPA, and XPC) and survival genes (e.g., Bcl-2, XIAP, and BirC5) (15). When considered together, these data suggested that strong ASNS expression might have contributed to tumor growth and EMT as well as contributed to cisplatin-sensitivity, probably through impacting NER expression as NER plays a significant role in repairing DNA damage induced by chemotherapeutic drugs (32, 33).

ASNS and autophagy play broad regulatory roles in asparagine homeostasis within tumor cells (34). In malignant KRAS-driven tumor cells, autophagy helped to reverse low asparagine-induced metabolic barriers and thus permitted tumor invasion (34). One might hypothesize that ASNS, asparaginase, and autophagy exist in a fine balance that maintains control of asparagine homeostasis in tumor cells. However, previous reports have not described the effect of ASNS on autophagy. Although we observed the ASNS-mediated inhibition of autophagy in both CS and CR cells, the levels of both ASNS expression and autophagy were lower in the latter cell type. This result may have been attributable to the high level of FLNA expression in CR cells, as autophagy was attenuated by FLNA overexpression but enhanced by FLNA knockdown. Moreover, we observed an increase in autophagy in CR cells subjected to both ASNS overexpression and FLNA knockdown, which was more consistent with the increased levels of autophagy in CS cells (higher ASNS, lower FLNA) than in CR cells (lower ASNS, higher FLNA).

FLNA, a key component of the TGF-β signaling pathway, is an important regulator of the EMT. This protein mediates cytoskeletal reorganization and is considered a potential marker of metastasis and poor prognosis (16, 17, 35, 36). However, other studies have shown that FLNA suppresses MMP activity and may attenuate the migration and invasion capacities of human fibrosarcoma cells (37). Furthermore, weak FLNA expression was shown to correlate with a poor prognosis in patients with nasopharyngeal, gastric, and renal cell carcinomas (35, 38). In this study, however, FLNA overexpression or knockdown did not affect cell proliferation, clonogenicity, invasion, migration, or EMT in CS or CR ovarian cancer cells. Nevertheless, we observed that the combined knockdown of FLNA and overexpression of ASNS enhanced all these processes when compared with ASNS overexpression alone. According to Yue et al., FLNA is involved in the repair of various types of DNA damage, including single- and double-strand breaks and inter-strand crosslinks, and defects of FLNA sensitized cancer cells to chemotherapeutic reagents (cisplatin and bleomycin) or ionizing radiation (39, 40). Therefore, FLNA-deficient cells may exhibit attenuated DNA repair processes that may accelerate the cell cycle and tumor growth, such as the upregulation of cell proliferation, clonogenicity, and EMT in CR cells with FLNA-knockdown and ASNS-overexpression when compared with CR cells with ASNS-overexpression alone.

In our study, FLNA overexpression promoted cisplatin resistance, whereas FLNA knockdown potentiated cisplatin-induced apoptosis. This outcome can be attributed to the essential role of FLNA in the efficient recombinational repair of DNA damage. Consequently, a reduction in FLNA expression sensitized cells to chemotherapy (39, 40). We further observed that FLNA overexpression inhibited autophagy while FLNA knockdown stimulated autophagy, and this suggested that this protein might protect against autophagy. Although ASNS overexpression suppressed autophagy, the addition of FLNA knockdown resulted in an increase in autophagy in CR cells. This increase might be attributable to inefficient DNA damage repair, given the potential effects of those genetic manipulations on NER gene expression and recombinational DNA repair.

In conclusion, our work not only generated a valuable proteomic profile of the differentially expressed proteins between LGSC and HGSC with corresponding clinical data, but also provided an insightful perspective regarding the potential functions of ASNS and FLNA with respect to cell proliferation, clonogenicity. and cisplatin resistance in HGSC and LGSC. These proteomics data might support the development of new diagnostic, prognostic, and chemotherapeutic strategies in ovarian cancer. In the future, the precise roles of ASNS and FLNA in HGSC and LGSC remain to be elucidated, and additional cellular models of these ovarian cancer subtypes are needed.

## Data Availability Statement

The datasets generated for this study can be found in the Supplementary Documents.

## Ethics Statement

The animal study was reviewed and approved by Institutional Animal Care and Use Committee of Guangzhou Medical University. Written informed consent was obtained from the individual(s) for the publication of any potentially identifiable images or data included in this article.

## Author Contributions

LZ, QW, and KS conceived and designed the experiments and the methodology. QW performed the *in vitro* experiments, contributed to the data analyses, and drafted the manuscript. LZ, CG, LY, XX, LH, and KC perpared the samples for iTRAQ analysis and performed the verification of protein expression by IHC staining. LZ, PT, LX, and SH recruited the patients and collected the HGSC and the LGSC tissues. LZ and KS contributed to the editing of the manuscript and contributed to the funds for this study.

### Conflict of Interest

The authors declare that the research was conducted in the absence of any commercial or financial relationships that could be construed as a potential conflict of interest.
