# Supplementary material for: Asparagine Synthetase and Filamin A Have Different Roles in Ovarian Cancer
Source: Front Oncol. 2019 Oct 18;9:1072. doi: 10.3389/fonc.2019.01072 (PMC6813569; doi:10.3389/fonc.2019.01072)
Supplement: Supplementary file 6 [file Table_6.DOC]

**Table S6** List of top 50 up-regulated proteins in HGSC groups

| **Protein name** | **Accession number** | **sequence coverage** | **molecular weight** | **Isoelectric point** | **Change**  **ratio** |
| --- | --- | --- | --- | --- | --- |
| Interferon-induced GTP-binding protein Mx1 | sp|P20591|MX1_HUMAN | 47.13 | 75.52 | 5.59 | 11.91 |
| Hydroxymethylglutaryl-CoA synthase, cytoplasmic | sp|Q01581|HMCS1_HUMAN | 15.77 | 57.29 | 5.21 | 8.43 |
| Guanylate-binding protein 1 | sp|P32455|GBP1_HUMAN | 44.76 | 67.93 | 5.97 | 7.44 |
| Signal transducer and activator of transcription 1-alpha/beta | sp|P42224|STAT1_HUMAN | 52.47 | 87.33 | 5.74 | 7.28 |
| DNA topoisomerase 2-alpha | sp|P11388|TOP2A_HUMAN | 27.47 | 174.38 | 8.82 | 6.45 |
| Stathmin | sp|P16949|STMN1_HUMAN | 71.81 | 17.3 | 5.76 | 6.33 |
| DNA replication licensing factor MCM2 | sp|P49736|MCM2_HUMAN | 31.53 | 101.89 | 5.34 | 6.3 |
| Proliferation marker protein Ki-67 | sp|P46013|KI67_HUMAN | 18.94 | 358.69 | 9.48 | 6.13 |
| Ferritin light chain | sp|P02792|FRIL_HUMAN | 60.86 | 20.02 | 5.51 | 6.13 |
| Galectin-4 | sp|P56470|LEG4_HUMAN | 21.21 | 35.94 | 9.21 | 5.82 |
| Carbonic anhydrase 1 | sp|P00915|CAH1_HUMAN | 75.29 | 28.87 | 6.59 | 5.78 |
| Hemoglobin subunit delta | sp|P02042|HBD_HUMAN | 99.32 | 16.05 | 7.84 | 5.77 |
| Amine oxidase [flavin-containing] A | sp|P21397|AOFA_HUMAN | 39.19 | 59.68 | 7.94 | 5.68 |
| ***Asparagine synthetase [glutamine-hydrolyzing]** | sp|P08243|ASNS_HUMAN | 24.6 | 64.37 | 6.39 | 5.59 |
| Spectrin beta chain, erythrocytic | sp|P11277|SPTB1_HUMAN | 41.18 | 246.46 | 5.14 | 5.42 |
| DNA replication licensing factor MCM7 | sp|P33993|MCM7_HUMAN | 31.44 | 81.31 | 6.08 | 5.34 |
| HLA class II histocompatibility antigen, DRB1-12 beta chain | sp|Q95IE3|2B1C_HUMAN | 40.6 | 29.88 | 7.69 | 5.24 |
| Antigen peptide transporter 2 | sp|Q03519|TAP2_HUMAN | 24.49 | 75.66 | 8.24 | 5.19 |
| dCTP pyrophosphatase 1 | sp|Q9H773|DCTP1_HUMAN | 44.12 | 18.68 | 4.93 | 5.19 |
| Proliferating cell nuclear antigen | sp|P12004|PCNA_HUMAN | 43.68 | 28.77 | 4.57 | 5.03 |
| Tryptophan--tRNA ligase, cytoplasmic | sp|P23381|SYWC_HUMAN | 49.9 | 53.16 | 5.83 | 4.97 |
| Probable ATP-dependent RNA helicase DDX58 | sp|O95786|DDX58_HUMAN | 30.65 | 106.59 | 6.03 | 4.55 |
| Nuclear autoantigenic sperm protein | sp|P49321|NASP_HUMAN | 32.68 | 85.24 | 4.26 | 4.4 |
| DNA replication licensing factor MCM3 | sp|P25205|MCM3_HUMAN | 31 | 90.98 | 5.53 | 4.15 |
| Interferon-induced protein with tetratricopeptide repeats 1 | sp|P09914|IFIT1_HUMAN | 39.75 | 55.36 | 6.75 | 4.11 |
| Structural maintenance of chromosomes protein 2 | sp|O95347|SMC2_HUMAN | 27.95 | 135.65 | 8.54 | 4.08 |
| Cellular retinoic acid-binding protein 2 | sp|P29373|RABP2_HUMAN | 67.76 | 15.69 | 5.42 | 3.83 |
| Kunitz-type protease inhibitor 2 | sp|O43291|SPIT2_HUMAN | 17.86 | 28.23 | 8.68 | 3.81 |
| E3 ubiquitin-protein ligase RNF213 | sp|Q63HN8|RN213_HUMAN | 22.13 | 591.4 | 6.05 | 3.79 |
| Phosphoserine aminotransferase | sp|Q9Y617|SERC_HUMAN | 40 | 40.42 | 7.56 | 3.75 |
| 2'-5'-oligoadenylate synthase 3 | sp|Q9Y6K5|OAS3_HUMAN | 26.87 | 121.17 | 8.73 | 3.73 |
| Probable ATP-dependent RNA helicase DDX60 | sp|Q8IY21|DDX60_HUMAN | 20.83 | 197.85 | 7.52 | 3.71 |
| Proteasome activator complex subunit 1 | sp|Q06323|PSME1_HUMAN | 77.11 | 28.72 | 5.78 | 3.68 |
| Alpha-(1,6)-fucosyltransferase | sp|Q9BYC5|FUT8_HUMAN | 27.13 | 66.51 | 7.36 | 3.65 |
| DNA replication licensing factor MCM5 | sp|P33992|MCM5_HUMAN | 31.2 | 82.28 | 8.64 | 3.59 |
| WD repeat and HMG-box DNA-binding protein 1 | sp|O75717|WDHD1_HUMAN | 14.26 | 125.96 | 5.41 | 3.56 |
| Spectrin alpha chain, erythrocytic 1 | sp|P02549|SPTA1_HUMAN | 38.14 | 280.01 | 4.95 | 3.55 |
| Ubiquitin-like protein ISG15 | sp|P05161|ISG15_HUMAN | 38.49 | 17.89 | 6.83 | 3.53 |
| Ena/VASP-like protein | sp|Q9UI08|EVL_HUMAN | 29.45 | 44.62 | 8.91 | 3.52 |
| Prostaglandin G/H synthase 1 | sp|P23219|PGH1_HUMAN | 24.96 | 68.68 | 6.81 | 3.51 |
| Band 3 anion transport protein | sp|P02730|B3AT_HUMAN | 36.5 | 101.79 | 5.07 | 3.5 |
| Transferrin receptor protein 1 | sp|P02786|TFR1_HUMAN | 37.44 | 84.87 | 6.18 | 3.5 |
| Cyclin-dependent kinase 1 | sp|P06493|CDK1_HUMAN | 50.17 | 34.09 | 8.37 | 3.23 |
| Fatty acid-binding protein, adipocyte | sp|P15090|FABP4_HUMAN | 35.61 | 14.72 | 6.59 | 3.23 |
| Structural maintenance of chromosomes protein 4 | sp|Q9NTJ3|SMC4_HUMAN | 30.01 | 147.18 | 6.37 | 3.23 |
| Protein RCC2 | sp|Q9P258|RCC2_HUMAN | 47.8 | 56.09 | 9.01 | 3.21 |
| Bisphosphoglycerate mutase | sp|P07738|PMGE_HUMAN | 49.03 | 30 | 6.1 | 3.2 |
| Delta(24)-sterol reductase | sp|Q15392|DHC24_HUMAN | 21.03 | 60.1 | 8.42 | 3.19 |
| Paralemmin-3 | sp|A6NDB9|PALM3_HUMAN | 15.23 | 71.69 | 4.52 | 3.12 |
| Flavin reductase (NADPH) | sp|P30043|BLVRB_HUMAN | 73.3 | 22.12 | 7.13 | 3.11 |
