# Supplementary material for: Asparagine Synthetase and Filamin A Have Different Roles in Ovarian Cancer
Source: Front Oncol. 2019 Oct 18;9:1072. doi: 10.3389/fonc.2019.01072 (PMC6813569; doi:10.3389/fonc.2019.01072)
Supplement: Supplementary file 7 [file Table_7.DOC]

**Table S7** List of top 50 down-regulated proteins in HGSC groups.

| **Protein name** | **Accession number** | **sequence coverage** | **molecular weight** | **Isoelectric point** | **Change**  **ratio** |
| --- | --- | --- | --- | --- | --- |
| Serpin B3 | sp|P29508|SPB3_HUMAN | 22.56 | 44.56 | 6.35 | 0.03 |
| Serotransferrin | sp|P02787|TRFE_HUMAN | 81.09 | 77.06 | 6.81 | 0.05 |
| Annexin A2 | sp|P07355|ANXA2_HUMAN | 89.97 | 38.6 | 7.57 | 0.05 |
| Immunoglobulin heavy constant gamma 2 | sp|P01859|IGHG2_HUMAN | 76.3 | 35.9 | 7.66 | 0.05 |
| Keratin, type I cytoskeletal 16 | sp|P08779|K1C16_HUMAN | 44.72 | 51.27 | 4.98 | 0.05 |
| Tenascin-X | sp|P22105|TENX_HUMAN | 36.89 | 458.21 | 5.05 | 0.06 |
| Mucin-5B O | sp|Q9HC84|MUC5B_HUMAN | 12.17 | 596.33 | 6.19 | 0.06 |
| Tubulin polymerization-promoting | sp|Q9BW30|TPPP3_HUMAN | 59.09 | 18.98 | 9.19 | 0.06 |
| protein family member 3 Neutrophil gelatinase-associated lipocalin | sp|P80188|NGAL_HUMAN | 77.28 | 22.59 | 9.02 | 0.07 |
| Cysteine and glycine-rich protein 1 | sp|P21291|CSRP1_HUMAN | 76.17 | 20.57 | 8.9 | 0.08 |
| Prelamin-A/C | sp|P02545|LMNA_HUMAN | 78.47 | 74.14 | 6.57 | 0.09 |
| IgGFc-binding protein | sp|Q9Y6R7|FCGBP_HUMAN | 12.92 | 572.01 | 5.14 | 0.09 |
| Keratin, type I cytoskeletal 23 | sp|Q9C075|K1C23_HUMAN | 38.63 | 48.13 | 6.09 | 0.09 |
| Keratin, type II cytoskeletal 5 | sp|P13647|K2C5_HUMAN | 39.66 | 62.38 | 7.58 | 0.09 |
| Protein S100-A6 | sp|P06703|S10A6_HUMAN | 83.34 | 10.18 | 5.32 | 0.1 |
| Keratin, type II cytoskeletal 6A | sp|P02538|K2C6A_HUMAN | 47.16 | 60.04 | 8.09 | 0.1 |
| Keratin, type I cytoskeletal 17 | sp|Q04695|K1C17_HUMAN | 62.5 | 48.1 | 4.97 | 0.1 |
| Deleted in malignant brain tumors 1 protein | sp|Q9UGM3|DMBT1_HUMAN | 15.67 | 260.73 | 5.18 | 0.1 |
| Putative ciliary rootlet coiled-coil protein 2 | sp|H7BZ55|CRCC2_HUMAN | 47.83 | 185.87 | 5.42 | 0.11 |
| Zinc finger protein 185 | sp|O15231|ZN185_HUMAN | 23.3 | 73.52 | 6.67 | 0.11 |
| Tropomyosin beta chain | sp|P07951|TPM2_HUMAN | 71.83 | 32.85 | 4.66 | 0.11 |
| ***Filamin-A** | sp|P21333|FLNA_HUMAN | 73.37 | 280.73 | 5.7 | 0.12 |
| Alpha-1-antitrypsin | sp|P01009|A1AT_HUMAN | 82.54 | 46.73 | 5.37 | 0.12 |
| Antithrombin-III | sp|P01008|ANT3_HUMAN | 65.84 | 52.6 | 6.32 | 0.12 |
| Collagen alpha-1(XIV) chain | sp|Q05707|COEA1_HUMAN | 61.03 | 193.51 | 5.16 | 0.13 |
| Periplakin | sp|O60437|PEPL_HUMAN | 48.21 | 204.74 | 5.47 | 0.13 |
| Complement factor H | sp|P08603|CFAH_HUMAN | 39.81 | 139.09 | 6.21 | 0.13 |
| Myosin-11 | sp|P35749|MYH11_HUMAN | 73.91 | 227.34 | 5.42 | 0.14 |
| Band 4.1-like protein 2 | sp|O43491|E41L2_HUMAN | 56.21 | 112.59 | 5.34 | 0.14 |
| Lactotransferrin | sp|P02788|TRFL_HUMAN | 60.5 | 78.18 | 8.5 | 0.14 |
| Alpha-1-acid glycoprotein 1 | sp|P02763|A1AG1_HUMAN | 57.46 | 23.51 | 4.93 | 0.14 |
| Neural cell adhesion molecule 1 | sp|P13591|NCAM1_HUMAN | 31.47 | 94.57 | 4.79 | 0.14 |
| Ceruloplasmin | sp|P00450|CERU_HUMAN | 49.72 | 122.2 | 5.44 | 0.15 |
| Annexin A1 | sp|P04083|ANXA1_HUMAN | 77.6 | 38.71 | 6.57 | 0.15 |
| Collagen alpha-3(VI) chain | sp|P12111|CO6A3_HUMAN | 63.61 | 343.66 | 6.26 | 0.16 |
| Collagen alpha-2(I) chain | sp|P08123|CO1A2_HUMAN | 75.96 | 129.31 | 9.08 | 0.16 |
| Alpha-2-macroglobulin | sp|P01023|A2MG_HUMAN | 61.33 | 163.29 | 6.03 | 0.16 |
| Transgelin | sp|Q01995|TAGL_HUMAN | 93.04 | 22.61 | 8.87 | 0.16 |
| Calcyphosin | sp|Q13938|CAYP1_HUMAN | 53.71 | 20.97 | 4.74 | 0.16 |
| Sushi domain-containing protein 2 | sp|Q9UGT4|SUSD2_HUMAN | 18.43 | 90.21 | 5.84 | 0.16 |
| 3-hydroxybutyrate dehydrogenase type 2 | sp|Q9BUT1|BDH2_HUMAN | 45.1 | 26.72 | 7.56 | 0.17 |
| Neuroblast differentiation-associated protein AHNAK | sp|Q09666|AHNK_HUMAN | 83.57 | 629.09 | 5.8 | 0.17 |
| Keratin, type II cytoskeletal 7 | sp|P08729|K2C7_HUMAN | 78.47 | 51.38 | 5.4 | 0.17 |
| Retinol-binding protein 1 | sp|P09455|RET1_HUMAN | 89.26 | 15.85 | 4.99 | 0.17 |
| Protein S100-A12 | sp|P80511|S10AC_HUMAN | 51.09 | 10.57 | 5.83 | 0.17 |
| G-protein coupled receptor family C group 5 member C | sp|Q9NQ84|GPC5C_HUMAN | 22 | 48.19 | 8.72 | 0.17 |
| Plectin | sp|Q15149|PLEC_HUMAN | 65.94 | 531.78 | 8.75 | 0.18 |
| Synaptopodin-2 | sp|Q9UMS6|SYNP2_HUMAN | 29.83 | 117.51 | 8.75 | 0.18 |
| Immunoglobulin heavy variable 5-51 | sp|A0A0C4DH38|HV551_HUMAN | 53.85 | 12.67 | 8.44 | 0.18 |
| Keratin, type I cytoskeletal 14 | sp|P02533|K1C14_HUMAN | 50.95 | 51.56 | 5.09 | 0.18 |
